# Options for monitoring and estimating historical carbon emissions from forest degradation in the context of REDD+

**DOI:** 10.1186/1750-0680-6-13

**Published:** 2011-11-24

**Authors:** Martin Herold, Rosa María Román-Cuesta, Danilo Mollicone, Yasumasa Hirata, Patrick Van Laake, Gregory P Asner, Carlos Souza, Margaret Skutsch, Valerio Avitabile, Ken MacDicken

**Affiliations:** 1 Wageningen University. Center for Geoinformation, Droevendaalsesteeg 3, 6708 PB Wageningen. The Netherlands; 2 UN-REDD Programme. FAO MRV team. Viale delle Terme di Caracalla 15, 00100 Rome. Italy; 3 Bureau of Climate Change, Forestry and Forest Products Research Institute. 1 Matsunosato, Tsukuba, 305-8687. Japan; 4 UN-REDD Vietnam Programme. 172 Ngoc Khanh, #805. Ba Dinh, Ha Noi. Vietnam; 5 Carnegie Institution. 260 Panama Street. Stanford, CA 94305. USA; 6 IMAZON, Rua Domingos Marreiros 2020, Fátima 66.060-160, Belém, Pará. Brazil; 7 Centro de Investigaciones en Geografía Ambiental, Universidad Nacional Autonoma de México, Campus Morelia, Antigua Carretera a Patzcuaro 8701, CP 58190, Morelia. México; 8 FAO Forest Resources Assessment team, Viale delle Terme di Caracalla 15. 00100 Rome. Italy

**Keywords:** REDD+, forest, global change, monitoring, deforestation, degradation, tropical countries, remote sensing

## Abstract

Measuring forest degradation and related forest carbon stock changes is more challenging than measuring deforestation since degradation implies changes in the structure of the forest and does not entail a change in land use, making it less easily detectable through remote sensing. Although we anticipate the use of the IPCC guidance under the United Framework Convention on Climate Change (UNFCCC), there is no one single method for monitoring forest degradation for the case of REDD+ policy. In this review paper we highlight that the choice depends upon a number of factors including the type of degradation, available historical data, capacities and resources, and the potentials and limitations of various measurement and monitoring approaches. Current degradation rates can be measured through field data (i.e. multi-date national forest inventories and permanent sample plot data, commercial forestry data sets, proxy data from domestic markets) and/or remote sensing data (i.e. direct mapping of canopy and forest structural changes or indirect mapping through modelling approaches), with the combination of techniques providing the best options. Developing countries frequently lack consistent historical field data for assessing past forest degradation, and so must rely more on remote sensing approaches mixed with current field assessments of carbon stock changes. Historical degradation estimates will have larger uncertainties as it will be difficult to determine their accuracy. However improving monitoring capacities for systematic forest degradation estimates today will help reduce uncertainties even for historical estimates.

## Introduction

From the perspective of the UNFCCC for REDD+, forest degradation refers to a loss of carbon stock within forest land. Forest disturbances that lead to degradation such as over-harvesting, forest fires, pests and climatic events including drought, wind, snow, ice, and floods have been estimated to affect roughly 100 million of hectares globally per year [1,2]. This value represents almost 10 times the area that is affected by deforestation globally (i.e. 13 million hayr^-1 ^for 2000-2005) [3,4]. In particular, tropical regions are well known for large scale disturbances that lead to forest degradation [5-8], but over large areas, the processes that reduce forest carbon stocks have neither been well characterized in space, nor in time.

To address climate change mitigation actions in the forest sector, five different components have been agreed upon by Parties to the United Framework Convention on Climate Change (UNFCCC) under negotiations for Reduced Emissions from Deforestation and Degradation (REDD+). These include reducing deforestation, reducing degradation, forest enhancement, sustainable management of forests, and forest conservation. The negotiations identify the need to establish national forest monitoring systems that use an appropriate combination of remote sensing and ground-based forest carbon inventory approaches for estimating anthropogenic forest-related greenhouse gas emissions by sources, removals by sinks, and the need to establish reference emission levels using historical data and adjusted for national circumstances [9].

Issues related to assessing and monitoring forest degradation and associated carbon stock changes have been subject to international debate on the political and technical level [10,11]. Recent history is of particular interest in the early stages of REDD+ implementation, in order to understand which drivers and activities have led to forest degradation and to quantify the carbon emissions caused by this process to provide a reference emission level. Because of the risk that action on deforestation may increase degradation, this is necessary to prove that REDD+ implementation has a positive impact [12].

Here we provide an overview of methods and approaches for monitoring carbon emissions from forest degradation, with a focus on historical periods. We structure the review around a set of critical issues and assumptions, as follows:

• REDD+ has specific monitoring requirements including a focus on the national level, the use of the IPCC guidance, the need to establish a reference emission level, and to assess how REDD+ policies and measures address the drivers and activities causing forest carbon loss,

• The IPCC guidance suggests the use of activity data (changes in extent of areas affected) and emission factors (changes in carbon stock within areas) to estimate emissions on the national level, with most effort to be put on the most important emission sources (i.e. key category analysis), and with different ways to handle uncertainties (i.e. different Tiers for carbon stock estimation), encouraging continuous improvements over time,

• Current and historical assessments of forest degradation need to be consistent, in order through serial correlation to reduce the impact of absolute uncertainty,

• Different methods including field measurement and remote sensing are needed to derive activity data and emission factors for different degradation processes. The data availability varies for differing historical periods and regions.

## Discussion

### Requirements for monitoring - definitions, drivers and the IPCC guidance

Equation 1 provides a conceptual overview of how to estimate gross carbon emission (Cgr_em) from forest land due to deforestation and loss of carbon stock in forest land remaining forest land, at the national level. Following the *Good Practice Guidance for Land Use, Land-Use Change and Forestry *(GPG-LULUCF) [13] and the Guidelines for Agriculture, Forestry and Other Land Use (AFOLU) [14], forest degradation uses methods to assess carbon stock changes in forest land remaining forest land, using a combination of activity data and emission factors. While deforestation usually removes almost all of the forest carbon stock permanently, the losses in term of carbon stock due to forest degradation depend on the type and the frequency of the human-induced disturbances. The equation demonstrates that the definition and distinction of deforestation and degradation need to be clear, and that different types of degradation processes exist.(1)

Forest degradation can be defined in many ways [15-17] but no single definition has been agreed upon at international level. Forest degradation, from the point of view of the UNFCCC for REDD+ purposes, refers to a loss of carbon stock within forest land that remain forest land [11]. The UNFCCC also refers to anthropogenic emissions and removals. Thus, we assume that degradation represents a human-induced negative impact on carbon stocks, with measured forest variables (i.e. canopy cover) remaining above the threshold for the definition of a forest. This threshold and other parameters vary from country to country but need to be applied consistently over time.

Besides the definition, in the REDD+ context it is necessary to understand the drivers and activities causing degradation. Such information is needed not only for formulating appropriate REDD+ strategies and policies, but also for the definition of suitable methods for measuring and monitoring. Various types of degradation will have different effects on the forest (carbon) and will result in different types of indicators (i.e. trees being removed, canopy damaged), which can be used for monitoring degradation using in situ and remote methods. Usually, different degradation processes are present within one country, with interactions among processes and recurrent events that leads to even more carbon emissions. Forest degradation processes may or may not affect large areas, but usually they are not equally distributed over the country's territory. They are often focused on specific areas, and this should be considered in national measurement and monitoring efforts [18,19].

The main drivers for direct forest degradation include:

a. ***Extraction of forest products for subsistence and local markets: ***privately or communally managed forests are often subject to extraction of forest products for immediate use or sale by local households, such as collection of fuelwood for cooking, collection of fruits, roots and other edible or medicinal tree parts, collection of fodder for livestock, and harvesting of timber and thatch for construction. In addition, most developing countries have seen rapid urbanization in recent decades, which has created a market for forest-based products (i.e. charcoal) that, in some cases, has resulted in forest degradation.

b. ***Industrial/commercial extraction of forest products: ***Large scale selective logging and other harvesting practices often occur in unregulated forest areas, exacerbated by poor logging practices such as multiple entries into forests [20].

c. ***Uncontrolled anthropogenic wildfire: ***This is a major source of degradation in many types of forests, and may be deliberate or accidental.

UNFCCC Decision 4/CP.15 [9] requests: "To use the most recent Intergovernmental Panel on Climate Change guidance and guidelines, as adopted or encouraged by the Conference of the Parties, as appropriate, as a basis for estimating anthropogenic forest-related greenhouse gas emissions by sources and removals by sinks, forest carbon stocks and forest area changes". In this context, countries should consider two measurement components to estimate the emissions associated with forest degradation:

1) Areas of forest that remain forest and are affected by degradation (considered at the national level), ideally stratified into different disturbances or degradation types. How much forest area, and where, is undergoing degradation? Such statistics, calculated through forest inventories or through remote sensing, are also referred to as Activity Data (AD). The GPG-LULUCF identifies three approaches to represent land areas, in increasing order of complexity [13]. For the assessment of forest degradation, only the most complex third approach seems most appropriate, where changes in land use categories can be tracked on a spatial basis [10].

2) Changes in forest carbon stocks due to the degradation processes per unit area. How much carbon is lost from the forests and released to the atmosphere due to the degradation process? Such amounts, commonly measured through forest field sampling and repeated forest inventories (and reported as MgCha^-1^yr^-1^) are also referred to as Emission Factors (EF). These changes should be calculated for each of the five forest carbon pools: aboveground biomass, belowground biomass, deadwood, litter, and soil organic matter [13]. The IPCC [13] provides three tiers for estimating emissions, with increasing levels of data requirements, analytical complexity and increasing accuracy. Tier 1 uses IPCC default values; Tier 2 uses country-specific data (i.e. collected within the national boundary) and Tier 3 uses actual inventories with repeated measurements to directly measure changes in forest biomass and/or well parameterized models in combination with plot data [10].

The IPCC guidelines [13] also provide the concept of key source categories that should be assessed and selected. A key source category is "an emission or sink category that is prioritized within the national inventory system because its estimate has a significant influence on a country's total inventory of direct greenhouse gases in terms of the absolute level of emissions, the trend in emissions, or both" [13]. Key source categories should be estimated using higher tiers where possible and thus help to focus the available monitoring resources on the most important components.

### Field observations and expert surveys to assess degradation

A critical step in estimating forest degradation is a well designed and implemented field sampling scheme to collect carbon stock data on the ground, in order to assess carbon stock changes over time. Field methods to evaluate carbon stock changes include [10]:

➣ Inventory-based approaches (national, sub-national),

➣ Data from targeted field surveys (including interviews) and from research and permanent sample plots, often implemented as local studies,

➣ Commercial forestry data (i.e. logging concessions and harvest estimates),

➣ Proxy data from domestic markets (charcoal, subsistence) such as timber production rates estimated from sawmill, sales, and export statistics [21].

If available, the collection of national forest data through periodic forest inventories since the 1980s allows the estimation of emissions associated with historical and current forest degradation processes [22]. When designing the sampling scheme of a National Forest Inventory, both the forest ecology and forest type are important in determining the expected biomass content and general properties of growth dynamics, and human practices that alter forest carbon, including degradation activities that reduce the carbon stock, need to be considered [23] and data collected stratified accordingly. Interactions between drivers, where significant, also need to be taken into account.

The estimation of forest carbon stock change with relatively low uncertainty (i.e. at Tier 3 level) assumes that consistent measurements are made at different points in time, i.e. before the degradation and at several points in time afterwards, to establish reliable emission factors. In most developing countries, however, the necessary long-term forest datasets are almost non-existent, or are focused on specific field assessments for commercial timber which cover only limited parts of the country. In these cases, the time variable has to be substituted by space (e.g. evaluating the net carbon stock decreases over a large area where all the successional stages of managed and unmanaged forests are present). This latter approach would consider the carbon stocks of intact and unmanaged forests as the reference value and by comparison would estimate the emissions of the degraded forests per unit of area.

Permanent sample plots are typically used to monitor changes in studies on forest resources and temporal dynamics. When historical records exist, it is worthwhile repeating measurements using the same sampling scheme. Forest inventory data are routinely collected by forestry organizations in many countries and are usually not focused on assessing the impact of forest degradation on carbon stocks. However, earlier inventories, for example those that focus on merchantable volumes of commercially interesting species, can be correlated with similar inventories in the present era, supplemented by information on forest properties that allows for the assessment of biomass, enabling an estimate of historical biomass content of the forest [24].

### Remote sensing methods to measure degradation

Measurement and monitoring of the area affected by forest degradation through remote sensing offers a series of advantages: i) it represents a consistent, coherent, transparent and fairly accurate way of reporting on area, and it allows for near-real time reporting on land use changes, ii) it offers spatially detailed national data even on remote and logistically complicated regions, and iii) it is the only approach that offers, potentially at least, objective information on historical trends in areas where data do not exist today. However, it also has several disadvantages: i) it can be hampered by clouds in some regions (for optical data), ii) it is limited by the technical capacity to sense and record the change in canopy cover (for fine-scale changes) and iii) image interpretations may be difficult equivocal and/or labor intensive, especially if national estimates are to be derived. Not all degradation processes can be monitored with high certainty using remote sensing data (Table 1). The more severe the degradation and the canopy damage, the easier it is to accurately map it from satellite observations [25]. Mapping from aircraft provides much more detail and resolves most of the limitations inherent to space-based measurements [26-28].

**Table 1:** Forest degradation activities and their degree of detection using Landsat-type data, adapted from [44].

Highly Detectable	Detection limited & increasing data/effort	Detection very limited
• Deforestation • Forest fragmentation • Recent slash-and-burn agriculture • Major canopy fires • Major roads • Conversion to tree monoculture • Hydroelectric dams and other forms of flood disturbances • Large-scale mining	• Selective logging • Forest surface fires • A range of edge-effects • Oldslash-and-burn agriculture • Small scale mining • Unpaved secondary roads (6-20 m wide) • Selective thinning of canopy trees	• Harvesting of most non-timber plants products • Low-mechanized selective logging • Narrow roads (< 6 m wide) • Understory thinning and clear cutting • Invasion of exotic species

Mapping forest degradation with remote sensing data is more challenging than mapping deforestation [29] because the degraded forest is a complex mix of different land cover types (vegetation, dead trees, soil, shade) and the signature of the degradation often changes within 1-2 years [30-32]. So far, to address forest degradation, medium spatial resolution sensors, such as Landsat, ASTER and SPOT, have mostly been used for degradation mapping. High and very high resolution satellite imagery, such as Ikonos or Quickbird, and aerial digital imagery acquired with videography have also been used. Methods for mapping forest degradation range from simple image interpretation to highly sophisticated automated algorithms [10].

With these issues in mind, there are three main approaches to evaluating forest degradation with remote sensing:

➣ Direct detection of degradation processes (observing forest canopy damage) and area changes, in which the features of interest to be enhanced and extracted from the satellite imagery consist of forest canopy gaps, small clearings and the structural forest changes resulting from disturbance [31,33,34]. This approach requires frequent mapping because the spatial signatures of the degraded forests change once canopy gaps close (i.e. gaps are covered by low-biomass secondary species).

➣ Indirect approaches (observing human infrastructure) are useful when degradation intensity is low (little canopy damage) or when the direct approach cannot be applied due to infrequent coverage and little spectral evidence remains from the canopy gaps. The remote sensing analysis focuses on the spatial distribution and evolution of human infrastructure (i.e. roads, population centers), which is used as a proxy for newly degraded areas [35,36]. This method works best to map newly degraded forest areas but is less effective for repeated degradation.

➣ Monitoring carbon emissions from biomass burning. This approach includes three primary categories: detection of active fires, mapping of post-fire burned areas (fire scars) and fire characterization (e.g. fire severity, energy released). For the purposes of emission estimation, the latter two categories, described in GOFC-GOLD (2010), are more relevant. The 'bottom up' method [37] uses the area affected by fire, the fuel loading per unit area, the proportion of biomass consumed as a result of fire (combustion factor) and the emission factor. A recently proposed alternative is directly to measure the power emitted by actively burning fires and to derive from this value the total biomass consumed [38,39]. However, this approach is less suitable for historical periods.

## Conclusions

Many developing countries will not have the data and capacities to provide suitable carbon emissions estimates on all types of forest degradation for historical periods [40]. Table 2 provides an overview of data source options for different degradation processes and drivers. Estimation of forest carbon changes in from historical degradation processes are unlikely to be able to rely on existing past data in many countries as there are little or no historical field data available. Remote sensing to establish extend and recent carbon density determination remains the only source to provide data for assessing past trends. This is particularly evident for degradation associated with local markets and subsistence, where the historical field data sources are generally rare and where remote sensing approaches have limited ability to provide information based on archived data. In this case, historical reference emission levels can hardly be established, particularly at the national level.

**Table 2:** Options for estimating activity data and emission factors for historical degradation on the national level beyond the use of default data (Tier 1).

Activity and driver of forest degradation	Suitable and available data sources for activity data (on national level)	Suitable and available data sources for emission factors (on national level)
Extraction of forest products for subsistence and local markets, such as fuelwood and charcoal	• Limited historical data • Information from local scale studies or national proxies (i.e. population growth and wood demand), if available • Only long-term cumulative changes may be observed from historical satellite data	• Limited historical data • Information from local scale studies, community-based monitoring or permanent sample plots, if available • Emission factors can be measured at present time and applied consistently for historical periods with suitable activity data

Industrial/commercial extraction of forest products such as selective logging	• Historical satellite data (Landsat time series) analysed with concession areas • Direct approach should be explored for recent years (i.e. since year circa-2000, depending on national coverage) and indirect approach for longer periods (back to 1990)	• National forest inventories and harvest estimates from commercial forestry (i.e. company records of wood volume extracted in selective logging activities in the past), if available • Emission factors can be measured today and can be applied consistently for historical periods with suitable activity data

Other disturbances such as (uncontrolled) wildfires	• Historical satellite-based fire data records (since 2000) to be analysed with Landsat-type data	• Emission factors can be measured today and can be applied consistently for historical periods with suitable activity data

Historical monitoring of industrial/commercial extraction of forest products can benefit from the use of archived satellite data, which could be analyzed with the support of other data sources such as forestry concession data. Specific emission factors can be estimated from present-day data on carbon stock losses due to similar degradation processes (i.e. as occurring at present) and by studying their chronosequences, applied consistently for historically periods with suitable activity data. In this case the estimation of historical reference emissions is driven by the activity data. A similar approach could be applied for the case of fires.

Table 2 is focused on the changes in the aboveground carbon pool, which is perhaps the most recognized and obvious carbon pool to estimate [41]. It is to be recognized that measuring the carbon stock changes caused by forest degradation in each pool within a country at consistent levels of detail and accuracy is unlikely to be possible. It may be advisable to focus monitoring on the most important categories (i.e. through an IPCC key source category analysis) and on specific areas within the country. This would help to make the monitoring more targeted and efficient, capturing the most important components [18,23]. In this context, there is a need to explore advanced approaches for spatial-temporal field sampling schemes, incorporating types of forest degradation by intensity and age, and integrating them with historical remote sensing data. In addition, we would also like to point out some examples on how uncertainties can be handled in a REDD+ implementation context [42,43].

## Competing interests

The authors declare that they have no competing interests.

## Authors' contributions

MH and RMRC led the review and the drafted the majority of the manuscript. DM, YH, PVL, GPA, CS, MS, VA and KMD conceived the study, added to the review and contributed to the manuscript. All authors read and approved the manuscript.
